# Perspectives on Drought Preconditioning Treatments With a Case Study Using Western Larch

**DOI:** 10.3389/fpls.2021.741027

**Published:** 2021-09-30

**Authors:** Ehren Reid Von Moler, Andrew Steven Nelson

**Affiliations:** Center for Forest Nursery and Seedling Research, College of Natural Resources, University of Idaho, Moscow, ID, United States

**Keywords:** biomass, drought preconditioning, root growth potential, seedling, water limitation, western larch, drought hardiness, *Larix occidentalis*

## Abstract

As the demand for drought hardy tree seedlings rises alongside global temperatures, there is a need to optimize nursery drought preconditioning methods to improve field performance of planted seedlings. This perspective article advocates for a more holistic approach to drought preconditioning research that considers the moderating role of plant developmental stage on the effects of drought preconditioning. We identify discrepancies in past studies of root growth potential (RGP) responses to drought preconditioning and highlight studies that suggest such discrepancies may result from inconsistencies among studies in the timing of drought preconditioning implementation. We then illustrate our perspective by presenting original research from an aeroponic RGP trial of 1st-year western larch (*Larix occidentalis* Nutt.) seedlings exposed to three soil moisture contents for 6months. We evaluated whether drought preconditioning could be used to increase the ratio of root: foliar tissue mass or enhance seedling physiological vigor during a subsequent growth period. Drought preconditioning was found to increase the ratio of root: foliar tissue mass and enhance seedling physiological vigor. Specifically, soil moisture content related negatively with new root biomass, positively with new foliar biomass, and negatively with the length and number of new roots (*p*<0.001). Meanwhile, the mass of lateral root production following drought preconditioning, but prior to aeroponic growth, correlated weakly to the mass, count, and length of new roots produced during aeroponic growth. We propose that evaluating the importance of the timing of drought preconditioning treatments constitutes an important research frontier in plant science.

## Introduction

Aridity in western North America threatens the survival of planted and natural tree seedlings (Vilagrosa et al., 2003; Minott and Kolb, 2020). Controlled exposure to water limitation in tree nurseries prior to outplanting (i.e., drought preconditioning) may increase seedling survival through altered seedling morphology and physiology while reducing nursery water consumption (Close et al., 2005; Grossnickle, 2012; Sloan et al., 2020). However, the literature does not consistently suggest that drought preconditioning promotes seedling traits associated with drought tolerance, and this may be due to a lack of consistency in drought preconditioning protocols. In this perspective article, we advocate for consistent, holistic approaches for conducting and evaluating the effects of drought preconditioning on tree seedlings, and we present results from a root growth potential (RGP) trial following drought preconditioning of western larch (*Larix occidentalis* Nutt.) seedlings. We draw attention especially to the need for greater investigation into how the timing of drought preconditioning may affect seedling responses, which, compared to the severity of water limitation, has received scant attention to date.

Assessments of RGP (a measure of plant physiological vigor that evaluates new root production after a short period of growth under optimal growth conditions in a controlled environment; Folk and Grossnickle, 1997) following drought preconditioning rapidly reveals whether water limitation treatments promote desirable belowground plant traits (Villar-Salvador et al., 2013). However, the literature shows little agreement among studies regarding the effects of drought preconditioning on RGP, and little consistency in the timing of drought preconditioning treatment initiation or duration. Furthermore, although plant developmental stage may moderate plant responses to stress (Zhou et al., 2016; Moler et al., 2021), it is unfortunately uncommon for investigators to explicitly state their reason for conducting drought preconditioning during a particular plant developmental period. For example, Villar-Salvador et al. (1999) subjected 6-month-old seedlings of Aleppo pine (*Pinus halapensis* Mill.) to three drought preconditioning regimes for 2months and concluded that drought significantly reduced RGP. Using a rapid dry-down technique consisting of 20min vs. 2h of root desiccation *via* exposure to air, Tinus (1996) found that RGP of 1-year-old Douglas-fir (*Pseudotsuga menziesii* var. glauca) seedlings decreased as the duration of root desiccation increased. In a unique multispecies comparison of three conifer species exposed to a treatment of varying water limitation intensities initiated after approximately 5months after seed sowing and lasting 2.5months, the RGP of white spruce [*Picea glauca* (Moench) Voss] increased with water limitation, but RGP of Douglas-fir [*Pseudotsuga menziesii* (Mirb.) Franco] and lodgepole pine (*Pinus contorta* Dougl.) was not affected (Driessche, 1992). Guarnaschelli et al. (2003) conducted a 36-day drought potted study using 5-month-old *Eucalyptus globulus* (Labill.) seedlings, and found that water-limitation reduced root mass compared to the high watering (control) treatment. And in a study of *Pinus pinea* L. exposed to 3months of water limitation using three watering levels initiated 4 months after seed sowing, Villar-Salvador et al. (2013) found that RGP decreased with the intensity of water limitation.

While short-term drought preconditioning treatments are common, evidence suggests that long-term treatments may be more effective at facilitating plant acclimation to drought (Duguy et al., 2013). For example, a study of three *Eucalyptus* spp. from xeric and riparian habitats showed that photosynthetic and hydraulic acclimation to drought conditions occurred after 4months of drought exposure but not after only 2months of drought exposure (Zhou et al., 2016). For many tree species that are regularly planted for reforestation, it remains unknown whether drought preconditioning might promote traits associated with drought hardiness. As climate change proceeds, however, progress in research on the improvement of plant drought hardiness is sorely needed (St. Clair and Howe, 2007), which we reflect upon through the following case study.

Western larch is a deciduous conifer species that occurs across the Inland and Pacific Northwest of North America, provides valuable timber and ecosystem services (Schmidt and Shearer, 1995), and is threatened by climate change (Rehfeldt and Jaquish, 2010). Drought preconditioning may improve establishment of planted western larch seedlings under dry conditions, but the effects of drought preconditioning of any duration on traits that promote establishment of western larch seedlings remain unknown. We addressed the following two research questions, which together may be used to assess whether drought preconditioning is effective at promoting seedling morphological traits associated with drought hardiness without impairing physiological vigor.

1. Does drought preconditioning increase the ratio of new root tissue mass fraction to new foliar tissue mass fraction?
2. Does drought preconditioning negatively affect seedling physiological vigor as expressed by shorter or fewer new roots?

## Materials and Methods

Seeds from six provenances across southeastern British Columbia, Canada and two half-sib improved families from a seed orchard in British Columbia representing seed sources in northwestern Montana, United States were used in this study. Western larch seedlings were grown in a greenhouse in 415C Styroblock® containers (Beaver Plastics, Alberta, Canada) with cavity volumes of 130ml. Seeds were sown by hand in Styroblock® container cavities filled with Berger© BM8 growing media (Saint-Modeste, QC, Canada) amended with 7.9g slow-release Osmocote® fertilizer (*N*=15%, *p*=9%, *K*=12%) per liter soil media. Berger© BM8 manufacturer specifications suggest the media has a water retention capacity ranging from 8 to 11 times the mass of a volume of soil. Following Campbell (n.d.), soil moisture retention curves were generated with a METER WP4C Dewpoint Potentiameter (METER Group, Inc. United States) to calculate that the Berger© BM8 media holds 0.462kg/kg gravimetric water content at the permanent wilting point (i.e., a soil-media water potential of −1.5MPa). Cavities were topped with TARGET® Forestry Sand (no. 9992002; Burnaby, BC, Canada) and thinned to one seedling per cavity as seedlings emerged. Beginning 6.5weeks after sowing, planted seedlings were subjected to 26weeks of drought preconditioning treatments (from June 1st to November 30, 2020) by holding plants at the following three gravimetric soil moisture contents: Low (50–65% saturated container weight), Medium (60–75% saturated container weight), and High (75–100% saturated container weight). Low, Medium, and High saturated container weights were calculated to correspond to the following respective ranges of gravimetric container water contents: 32–42, 39–48, and 48–65% (Dumroese et al., 2015). We initiated drought preconditioning treatments at the beginning of the rapid growth phase and maintained treatments through the seedling hardening phase. To establish saturated container weights, tray weights were measured after watering planted Styroblock® containers with a boom irrigation system until tray weight did not increase after further watering, and after gravitational water drained from trays for 2h (following MacDonald et al., 2012). New saturated container weights were calculated monthly to adjust for increases in seedling mass. Targeted percentages of saturated container weights were maintained by weighing containers daily and irrigating trays when tray weights reached the lowest weight permitted for a given soil moisture content treatment (e.g., 50% saturated container weight for the Low treatment). Each pass of the boom irrigation system increased tray weights by 5% of saturated container weight, which facilitated application of the volume of water required to restore tray gravimetric moisture contents to the maximum content permitted for each treatment. Container weights were based on the average weight across five trays per soil moisture treatment. Bud set was hastened using a 12-day reduced day-length treatment (10.5h light, 13.5h dark) applied 9.5weeks after initiating drought preconditioning. Average temperature in the greenhouse from the start of the experiment until the end of the short-day treatment was 21.7°C and average relative humidity was 66%. About 8months after seed sowing, seedlings were removed from containers following standard practices used at the University of Idaho Franklin H. Pitkin Forest Nursery, i.e., trays were secured in-place and seedlings were gently pulled from cavities by the base of seedling stems. Seedlings were then stored at −2.2°C for 1month prior to the RGP trial.

Twenty seedlings from each provenance × watering regime combination (*n*=480) were slowly thawed in a refrigerator at 2°C for 2days before gently washing roots free of soil media with tapwater. Rubber gaskets set into a plastic hanger were secured around seedling root collars to suspend roots atop misting chambers as described in Nelson (2019). Full spectrum LED light panels were suspended above chambers to a height at which seedlings were evenly illuminated with a photosynthetic photon flux density of 250μmolm^−2^ s^−1^ under a photoperiod of 14-h days and 10-h nights. Seedlings were grown in mist chambers for 21days before recording the following measurements: seedling height from root collar to the distal end of the terminal bud (cm), root collar diameter (mm) at the location where hypocotyl and root tissue differentiate visually, the number of new white root tips ≥1cm in length, and the length of the longest new root (cm), following Nelson (2019). New white lateral root tissue was separated from old (dark-toned) lateral root tissue with a razor and placed in coin envelopes to dry. Old lateral root tissue was separated from taproot tissue, and each tissue type was placed in coin envelopes to dry. All tissues were dried in a force-draft oven for a minimum of 72h at 65°C. Weights of each root tissue type and aboveground growth produced during the RGP trial, as well as weights of roots and woody stem and foliage prior to the RGP trial, were recorded to the nearest 10^−4^g using a Veritas® M214I balance (H & C Weighing Systems™).

Seedling root length and the ratio of new root mass fraction to new foliage mass fraction in response to drought following the first growth season were assessed using linear mixed-effect models (lme4 package in the *R* statistical environment, version 3.6.2). Count of new root tips was treated as an integer and analyzed specifying a Poisson distribution and log-link function in a generalized linear mixed-effects model (glmmTMB package in *R*). A Gaussian distribution was specified for models of all other response variables. For all models, provenance was included as a random effect and normality of model residuals was assessed using quantile-quantile plots. Tukey HSD was used to calculate *post hoc* contrasts. Estimated marginal means and corresponding CIs of new root count were back-transformed from log values derived from models following Strimbu et al. (2018). To investigate whether initial lateral root mass (i.e., following drought preconditioning but before aeroponic growth) influenced the production of roots during aeroponic growth, Pearson product–moment correlations were calculated for initial lateral root mass as a function of: (1) new lateral root mass, (2) new root tip count, and (3) length of the longest new root.

## Results

**Figure 1:**
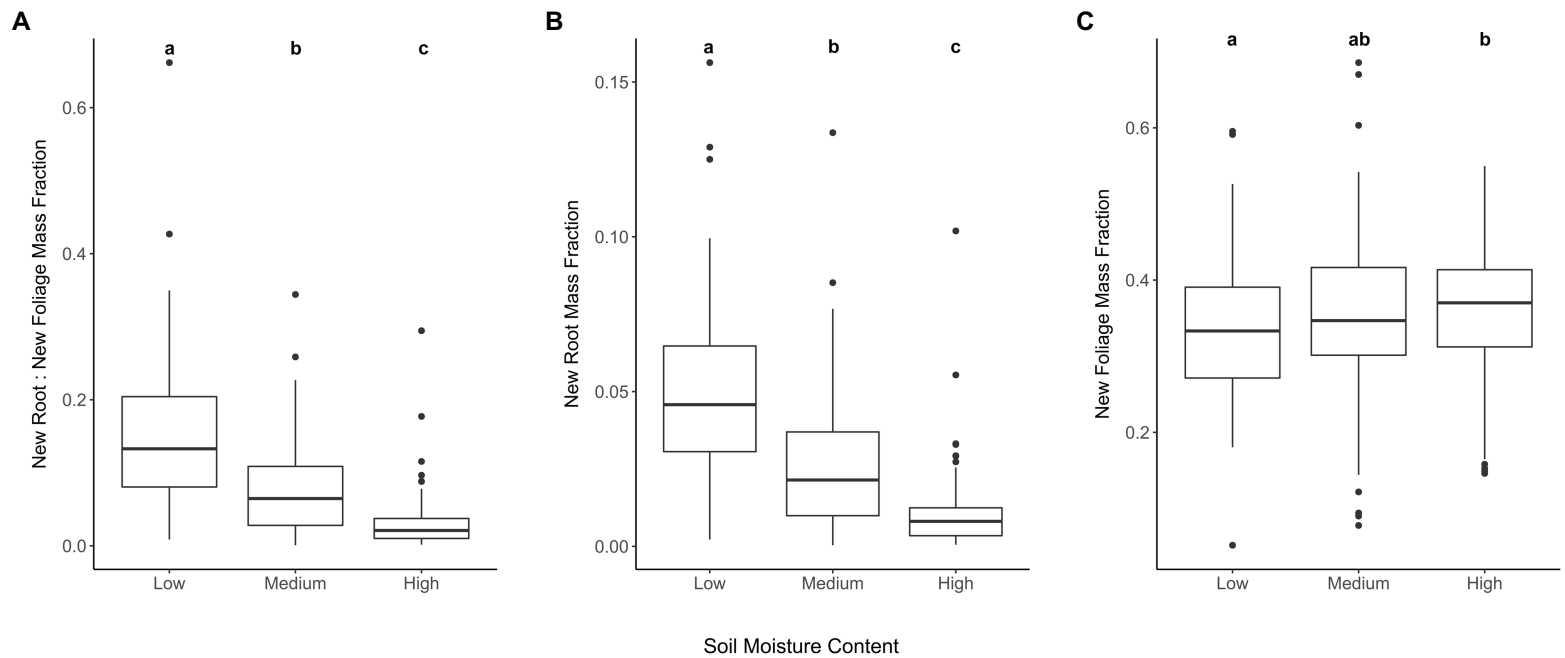
New root and foliage tissue biomass responses to drought preconditioning soil moisture contents. Ratio of new root to new foliar tissue biomass (A). Seedling root biomass (B) and foliage (C) produced during RGP trial standardized by total seedling biomass prior to RGP trial. Treatment levels that do not share lower-case letters within a panel are significantly different based on Tukey’s HSD pairwise comparisons (*p*<0.05).

The first research question was supported in that water limitation during long-term drought preconditioning increased the ratio of new root tissue to new foliar tissue mass (*χ*^2^=184.13, *p*=2.2e^−16^; Table 1A; Figure 1A). The largest proportional difference in new biomass attributed to new roots vs. new foliage occurred between the Medium and Low watering treatments (Table 1A). Water limitation during drought preconditioning correlated positively with new root mass and negatively with new foliage mass (Table 1B), though when corrected for initial total mass it is evident that root production responded more than foliage production to water limitation (Figures 1B,C). For each gram of initial lateral root biomass, seedlings in the Low, Medium, and High watering treatments produced a mean of 0.11, 0.07, and 0.04g of new root biomass, respectively. Meanwhile, lateral root masses at the end of the drought preconditioning period and prior to the RGP study were 0.77, 0.89, and 0.76 for seedlings subjected to the Low, Medium, and High watering treatments, respectively. A low but statistically significant correlation was found between initial lateral root mass and the mass of new roots produced during the RGP trial (*r*=0.13, *t*=2.5, *p*=0.012).

**Table 1:** **(A)** Estimated marginal means of treatment contrasts and **(B)** morphological measures by drought preconditioning treatment (±95% CI shown in parentheses).

A						
	New root: shoot mass fraction					
	Treatment contrast	*p*	Estimate	SE	*t*	
	Low - Medium	<0.0001	0.076	0.009	8.441	
	Low - High	<0.0001	0.121	0.009	13.423	
	Medium - High	<0.0001	0.045	0.009	5.011	
	Treatment	Emmean	lower.CL		upper.CL	
	Low	0.151	0.138	-	0.164	
	Medium	0.075	0.062	-	0.088	
	High	0.030	0.017	-	0.043	
	Count of new root tips					
	Treatment contrast	*p*	Estimate	SE	*t*	
	Low - Medium	<0.0001	0.169	0.016	10.870	
	Low - High	<0.0001	0.615	0.018	34.712	
	Medium - High	<0.0001	0.446	0.018	24.450	
	Treatment	Emmean	lower.CL		upper.CL	
	Low	56.990	53.671	-	59.912	
	Medium	48.080	45.280	-	51.053	
	High	30.657	28.872	-	32.553	
	Length of longest new root					
	Treatment contrast	*p*	Estimate	SE	*t*	
	Low - Medium	0.0014	1.670	0.477	3.511	
	Low - High	<0.0001	4.640	0.478	9.708	
	Medium - High	<0.0001	2.970	0.477	6.218	
	Treatment	Emmean	lower.CL		upper.CL	
	Low	11.600	10.780	-	12.430	
	Medium	9.930	9.110	-	10.750	
	High	6.960	6.130	-	7.790	
B						
Treatment	New foliage mass (g)		New root mass (g)	New root tip count		Longest new root length (cm)
Low	0.57 (±0.03)		0.08 (±0.01)	56.73 (±4.31)		11.60 (±0.65)
Medium	0.84 (±0.04)		0.06 (±0.01)	48.04 (±4.33)		9.93 (±0.76)
High	1.11 (±0.05)		0.03 (±0.01)	30.74 (±3.43)		6.96 (±0.59)

CL is 95% confidence limit; t is the calculated t statistic; p indicates the probability of observing the value of t by chance.

The second research question was not supported, i.e., water limitation corresponded to a greater abundance of new roots (*χ*^2^=1208.9, *p*<0.0001; Table 1A) and longer new roots (*χ*^2^=97.2, *p*<0.0001; Table 1A). The largest increase in root number and root length across levels of water limitation occurred between the High and Medium treatments (Table 1A), and water limitation correlated positively with both the number of new root tips and the length of the longest new roots (Table 1B). Low but statistically significant correlations were found between initial lateral root mass and root tip count (*r*=0.26, *t*=5.8, *p*=1.212e-08), and between initial lateral root mass and the length of the longest new root (*r*=0.13, *t*=2.9, *p*=0.003).

## Discussion

### Case Study

Long-term drought preconditioning of western larch seedlings promoted an increase in the production of new roots relative to new foliar tissue. Altered partitioning of carbon between root and shoot tissues associated with water limitation may result from responses of plant hormones (auxin, abscisic acid, gibberellin, and cytokinin) to drought (McAdam et al., 2016; Omena-Garcia et al., 2019; Sinclair and Friml, 2019; Ramachandran et al., 2020). Morphological shifts to more root vs. foliar tissue are expected to correspond to reduced seedling transplant shock and greater seedling outplanting survival due to greater root abundance facilitating enhanced uptake of soil water coupled with decreased foliar tissue and associated reductions in leaf-level evaporative demand (Close et al., 2005; Grossnickle, 2005). Furthermore, long-term drought preconditioning appears to have enhanced the physiological vigor of western larch seedlings, as water limitation increased the mass, count, and length of new roots produced during aeroponic growth (none of which were strongly correlated with initial lateral root mass). These results agreed with those of Pritzkow et al. (2021), who subjected seedlings of *Eucalyptus obliqua* to long-term drought preconditioning and found that preconditioning induced drought-adaptive reductions in foliar biomass. Pritzkow et al. (2021) reported that drought preconditioning did not influence plant water-relations or anatomy, but the smaller aboveground mass of preconditioned seedlings was associated with reduced water use and increased seedling survival under dry outplanting conditions. The present study established that long-term drought preconditioning of western larch promotes the development of seedling traits associated with establishment success in dry field conditions. Future studies of western larch should aim to establish whether a shorter-term preconditioning treatment initiated at an optimal plant developmental stage is capable of achieving similar desirable results in less time.

### Perspectives on Advancing Drought Preconditioning Research

Evidence suggests that the developmental period during which plants are exposed to abiotic stress can influence stress acclimation treatments, such as drought preconditioning. For example, in a study of the timing of greatest sensitivity to moderate moisture stress, Álvarez et al. (2013) found that geraniums (*Pelargonium*×*hortorum* L. H. Bailey) watered to 75% of container weight at field capacity during flowering were stunted in height and produced less flowers than plants subjected to moisture deficits outside of the flowering period. In a potted study consisting of 3-month-old and 1-year-old Aleppo pine (*P. halapensis* Mill) seedlings exposed to 39days of drought stress, Alexou (2013) found that seedling age significantly influenced ecophysiological and biochemical seedling responses to imposed drought. In a field study of *Pinus tabuliformis*, Luo et al. (2021) exposed 3-month-old seedlings to three drought hardening intensities fully crossed with three durations (2, 3, and 4weeks) of drought in the nursery. Seedlings exposed to the medium-duration drought treatment had the highest mortality but greatest growth in the field. Meanwhile, drought hardening intensity did not affect mortality and had a minor effect on seedling growth (Luo et al., 2021). In a study of southwestern white pine (*Pinus strobiformis* Engelm.) seedlings exposed to experimental warming during embryogenesis, seed germination, and early seedling growth, Moler et al. (2021) found that elevated temperatures during germination and early seedling growth, but not during embryogenesis, altered oxidative stress resistance, seedling morphology, and water relations physiology. Meanwhile, seedling survival was influenced by warming applied during all developmental stages. Given the historical prevalence of short- rather than long-term abiotic stress preconditioning and inconsistent selection of the developmental period during which preconditioning is initiated (e.g., when seedlings are rapidly growing vs. when seedling meristematic activity and growth slows during the hardening phase), the potential for drought preconditioning to improve plant drought hardiness remains untapped. The drought preconditioning treatment used in the present case study was initiated during the beginning of the rapid growth phase and was maintained through the hardening phase, and it remains unclear whether preconditioning during either developmental phase alone could have produced similar desirable seedling traits. Based on this and prior studies, we encourage future investigations into the potential for not only the severity but also the timing of drought preconditioning, including each seedling growth phase separately and a long-term treatment encompassing all phases, to induce desirable drought acclimation responses. Finally, while RGP trials provide an important and consistent measure of the effects of drought preconditioning on seedling performance, the efficacy of a drought preconditioning regime should also be assessed by measuring seedling survival through at least the second year of growth under dry field conditions, as illustrated by Benigno et al. (2014).

## Data Availability Statement

The datasets presented in this study can be found in online repositories. The names of the repository/repositories and accession number(s) can be found below: EM (2021, July 13). Timing is Everything: Long-Duration Drought Preconditioning Induces Drought-Hardy Traits in Western Larch (*Larix occidentalis*) Seedlings (Retrieved from osf.io/jer72).

## Author Contributions

EM and AN designed the study, grew the experimental seedlings, and conducted measurements. EM analyzed the data and drafted the manuscript. AN provided critical reviews and coordinated funding that facilitated this project. All authors contributed to the article and approved the submitted version.

## Funding

This work was funded by the United States Department of Agriculture, National Institute of Food and Agriculture grant 2019-67014-29109 and the National Science Foundation award 1916699. Publication of this article was funded by the University of Idaho – Open Access Publishing Fund.

## Conflict of Interest

The authors declare that the research was conducted in the absence of any commercial or financial relationships that could be construed as a potential conflict of interest.

## Publisher’s Note

All claims expressed in this article are solely those of the authors and do not necessarily represent those of their affiliated organizations, or those of the publisher, the editors and the reviewers. Any product that may be evaluated in this article, or claim that may be made by its manufacturer, is not guaranteed or endorsed by the publisher.
